# Supplementary material for: Biomechanics and neural circuits for vestibular-induced fine postural control in larval zebrafish
Source: Nat Commun. 2023 Mar 10;14:1217. doi: 10.1038/s41467-023-36682-y (PMC10006170; doi:10.1038/s41467-023-36682-y)
Supplement: Supplementary file 2 — Supplementary Information [file 41467_2023_36682_MOESM2_ESM.pdf]

Supplementary Information

Biomechanics and neural circuits for vestibular-induced fine postural control in larval zebrafish

Takumi Sugioka[1,2,3], Masashi Tanimoto[1,2,3,4,*] and Shin-ichi Higashijima[1,2,3,4,5,*]

[1]Exploratory Research Center on Life and Living Systems, Okazaki, Aichi 444-8787, Japan

[2]National Institute for Basic Biology, Okazaki, Aichi 444-8787, Japan

[3]The Graduate University for Advanced Studies, SOKENDAI, Okazaki, Aichi 444-8787, Japan

[4]These authors jointly supervised this work

[5]Lead contact

[*]Correspondence: tanimoto@nibb.ac.jp; shigashi@nibb.ac.jp

Supplementary Table 1. List of transgenic lines

Transgenic lines	Source
Tg(*evx2*-hs:GFP)<br>noted as Tg(*evx2*:GFP) in the text	Kawano *et al.*, 2022[1]
Tg(*evx2*-hs:Gal4)<br>noted as Tg(*evx2*:Gal4) in the text	Kimura *et al.*, 2014[2]
Tg(UAS:Dendra2)	Taniguchi *et al.*, 2017[3]
Tg(*vglut1*-hs:loxP-mCherry-loxP-Gal4)<br>noted as Tg(*vglut1*:RFP) in the text	This paper
Tg(*vglut2a*:loxP-RFP-loxP-GFP)<br>noted as Tg(*vglut2a*:RFP) in the text	Satou *et al.*, 2012[4]
Tg(*glyt2*-hs:loxP-RFP-loxP-GFP)<br>noted as Tg(*glyt2*:RFP) in the text	Satou *et al.*, 2020[5]
Tg(*gad1b*:loxP-RFP-loxP-GFP)<br>noted as Tg(*gad1b*:RFP) in the text	Satou *et al.*, 2013[6]
Tg(*evx2*-hs:tdTomato-jGCaMP7b)<br>noted as Tg(*evx2*:tdTomato-jGCaMP7b) in the text	This paper
Tg(*evx2*-hs:CoChR-GFP-Kv2.1)<br>noted as Tg(*evx2*:CoChR-GFP) in the text	This paper
Tg(*pitx2*-hs:Dendra2)<br>noted as Tg(*pitx2*:Dendra2) in the text	This paper
Tg(*smyhc2*-hs:tdTomato-jGCaMP7b)<br>noted as Tg(*smyhc2*:tdTomato-jGCaMP7b) in the text	This paper
Tg(*tbx2b*-hs:Cre)<br>noted as Tg(*tbx2b*:Cre) in the text	This paper
Tg(*smyhc2*-hs:loxP-RFP-loxP-DTA)<br>noted as Tg(*smyhc2*:loxP-RFP-loxP-DTA) in the text	This paper
Tg(*α-actin*:GFP)	Higashijima *et al.*, 1997[7]
Tg(*α-actin*:tdTomato-jGCaMP7f)	This paper
Tg(*vachta*-hs:Gal4)<br>noted as Tg(*vachta*:Gal4) in the text	Taniguchi *et al.*, 2017[3]
Tg(UAS:Kaede)	Hatta *et al.*, 2006[8]

Supplementary Table 2. List of key reagents

Antibodies	Source	Identifier
S58	DSHB	Cat# s58, RRID:AB_528377
Goat anti-Mouse IgG (H+L) Highly Cross-Adsorbed Secondary Antibody, Alexa Fluor 488	Thermo Fisher Scientific	Cat# A-11029, RRID:AB_2534088
**Chemicals**	**Source**	**Identifier**
Ethyl 3-aminobenzoate methanesulfonate salt (MS-222)	Sigma Aldrich	Cat# A5040
Methylcellulose	Sigma Aldrich	Cat# A0387
Agarose-LM	nacalai tesque	Cat# 01161-12
Sucrose	Wako	Cat# 196-00015
Cal-520 Dextran conjugate MW 10,000	AAT bioquest	Cat# 20601
Dextran, Tetramethylrhodamine, 3000 MW, Anionic, Lysine Fixable	Thermo Fisher Scientific	Cat# D3308

Supplementary Table 3. List of key optomechanical components

Optomechanical components	Source	Product identifier
Motorized rotation stage	Thorlabs	HDR50/M
Motorized rotation stage	Thorlabs	DDR100/M
Light source	Excelitas Technologies	X-Cite exacte
Episcopic illuminator	Olympus	BX-URA2
Tube lens unit	Olympus	U-TR30-2
C-mount camera adaptor	Olympus	U-TV0.63XC
Image splitting optics	Hamamatsu Photonics	W-VIEW GEMINI, A12801-01
Digital camera	Hamamatsu Photonics	ORCA-Flash4.0 V3, C13440-20CU
Digital camera	Teledyne FLIR	GS3-U3-23S6M-C
Digital camera	Basler	acA640-750um

Supplementary Table 4. List of key software and algorithms

Software and algorithms	Version	Source	URL
Kinesis	1.14.23	Thorlabs	https://www.thorlabs.com/newgrouppage9.cfm?objectgroup_id=10285
FlyCature2	2.13.3.61	Teledyne FLIR	https://flycap2-viewer-release.software.informer.com
Pylon Viewer	6.2.0.21487	Basler	https://www.baslerweb.com/en/products/basler-pylon-camera-software-suite/
HC Image Live	4.4.0.11	Hamamatsu	https://hcimage.com/hcimage-overview/hcimage-live/
LAS X	3.5.7.2325	Leica microsystems	https://www.leica-microsystems.com/products/microscope-software/p/leica-las-x-ls/
ImageJ/Fiji	1.53t (ImageJ)<br>2.9.0 (Fiji)	NIH	https://imagej.net<br>https://fiji.sc
Template Matching and Slice Alignment Plugin	#2015/2/07	Tseng Qingzong	https://sites.google.com/site/qingzongtseng/template-matching-ij-plugin
R	4.1.2	R Core Team	https://www.r-project.org/
Excel	16.65	Microsoft	https://www.microsoft.com/en-us/microsoft-365/excel

Supplementary Figure 1

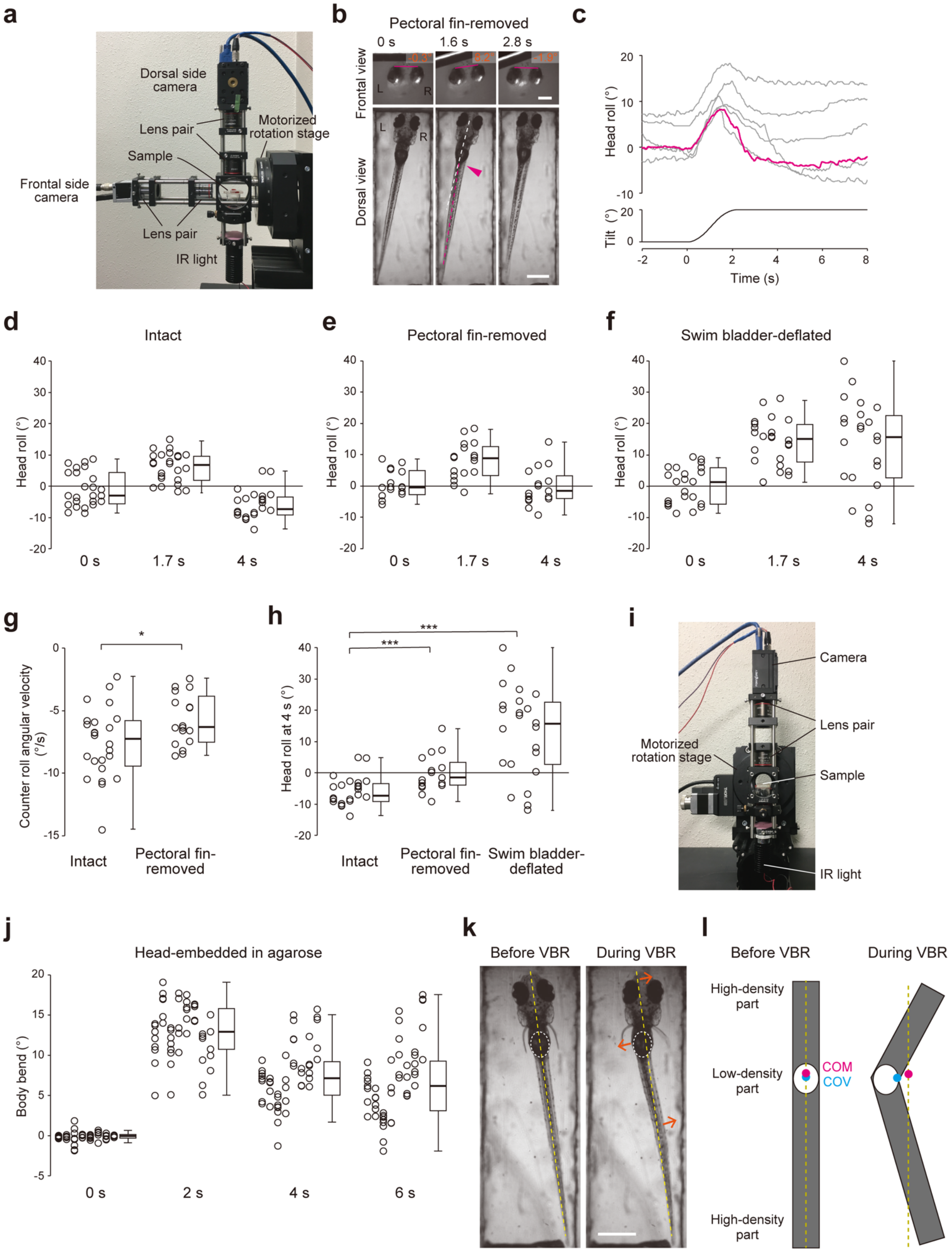

Supplementary Figure 1. Behavior of fin-removed fish and population data from behavioral experiments

a Lateral view photograph of the experimental setup for filming fish behaviors upon roll-tilt stimuli.

b, c Behavioral experiments on pectoral fin-removed fish. **b**: Snapshots of the frontal and dorsal images of a fish during a left-down tilt. **c**: Traces of the head roll angles of a fish in response to roll tilt. The magenta trace corresponds to the trial shown in **b**.

d–**f** Population data for the head roll angles of intact (**d**; 24 trials from five fish), pectoral fin-removed (**e**; 17 trials from three fish), and swim bladder-deflated fish (**f**; 23 trials from five fish). The head roll angles for each trial were measured at 0, 1.7, and 4 s from the onset of roll-tilt stimulus. Three to six trials were conducted for each fish. Data obtained from same fish are vertically aligned.

g Population data for the counter-roll angular velocity of the head. Box plots are shown on the right. This is the same fish as in **d** and **e**. $p = 0.04$ (two-sided Wilcoxon rank-sum test)

h Comparisons of head roll angles at 4 s. The same data as in **d**–**f**. $p = 0.0005$ (two-sided Wilcoxon rank-sum test) for intact vs. pectoral fin-removed, $p = 4.8 \times 10^{-6}$ (two-sided Wilcoxon rank-sum test) for intact vs. swim bladder-deflated.

i Frontal view photo of the experimental setup for filming fish behaviors in the head-embedded condition upon roll-tilt stimuli.

j Population data of the body bend angles in head-embedded fish. The body bend angles for each trial were measured at 0, 2, 4, and 6 s from the onset of the roll-tilt stimulus. Forty-nine trials from eight fish (five to eight trials for each fish) are shown. Data obtained from the same fish are vertically aligned. Box plots are shown on the right.

k Body displacement caused by the VBR. Snapshots of the fish before and during the VBR. The fish images are the same as those in Figure 1d. In both images, yellow dashed lines denote the fish midline before the VBR. White dashed circles indicate the swim bladders. Orange arrows show displacement of the corresponding body regions. The head and caudal body move toward the contraction side while the body around the swim bladder moves to the stretched side.

l Simplified schematic of body displacement caused by the VBR. Yellow dashed lines denote the midline before the VBR. Magenta and cyan circles indicate the center of mass (COM) and center of volume (COV), respectively. The COM position in the absolute coordinate does not change during the VBR, while the COV position moves to the stretched side.

Box plots in **d**–**h**, **j:** Boxes represent the interquartile range (IQR) between the first and third quartiles and the line inside represents the median. Whiskers denote the lowest and highest values within 1.5 × IQR from the first and third quartiles, respectively.

Scale bars, frontal images in **b** 200 μm; dorsal images in **b**, **k** 500 μm

Source data are provided as a Source Data file.

Supplementary Figure 2

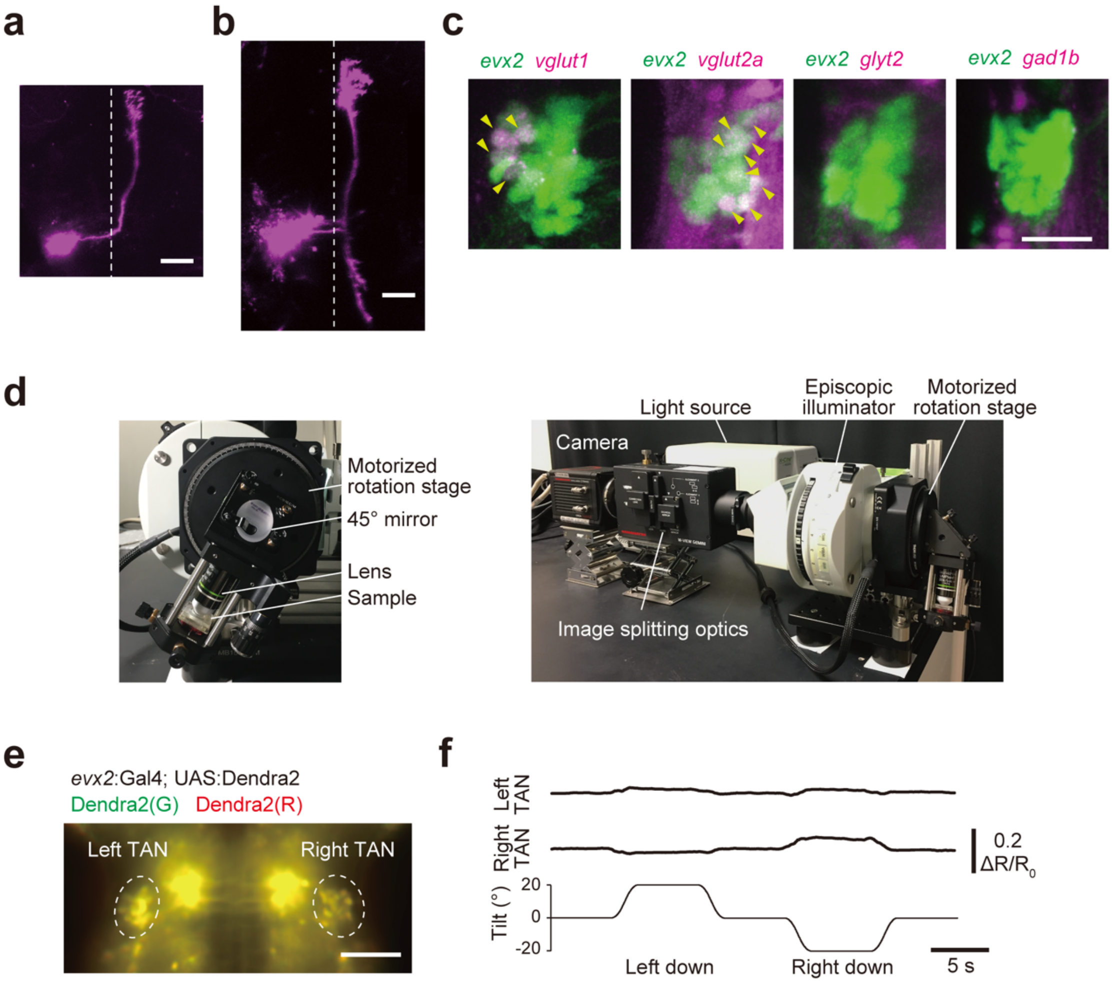

Supplementary Figure 2. Characteristics of TAN neurons and the performance of the widefield-version of the tiltable objective microscope

a, **b** Single-cell morphologies of TAN neurons revealed by electroporation of rhodamine-dye. Maximum intensity projection of confocal stacks of dorsal view images. Dashed lines indicate the midlines. Rostral is to the top. Lateral is to the left. **a**: A TAN neuron with an ascending axon. Similar data was observed in 2 fish. **b**: A TAN neuron with a bifurcating (ascending/descending) axon. Similar data was observed in 6 fish.

c Neurotransmitters properties of *evx2*-positive TAN neurons, as revealed by crossing Tg(*evx2*:GFP) to RFP-expressing marker transgenic fish lines for the following neurotransmitter phenotypes: Tg(*vglut1*:RFP), Tg(*vglut2a*:RFP), Tg(*glyt2*:RFP), and Tg(*gad1b*:RFP). *vglut1* and *vglut2a* are markers for glutamatergic neurons, *glyt2* is a marker for glycinergic neurons, and *gad1b* is a marker for GABAergic neurons. Yellow arrowheads show the overlap of fluorescence. Dorsal views of confocal stacked images.

d Frontal (left) and lateral (right) views of the widefield-version (non-confocal-version) of the tiltable objective microscope for Ca^{2+} imaging of TAN neurons during roll tilts.

e Image (merge of the green and red channels) of Tg(*evx2*:Gal4; UAS:Dendra2) after a partial photoconversion of Dendra2.

f Time courses of $\Delta R/R_0$ in the left and the right TAN neurons in response to a roll tilt.

Scale bars, **a**, **b**, **e** 50 μm; **c** 20 μm

Source data are provided as a Source Data file.

Supplementary figure 3

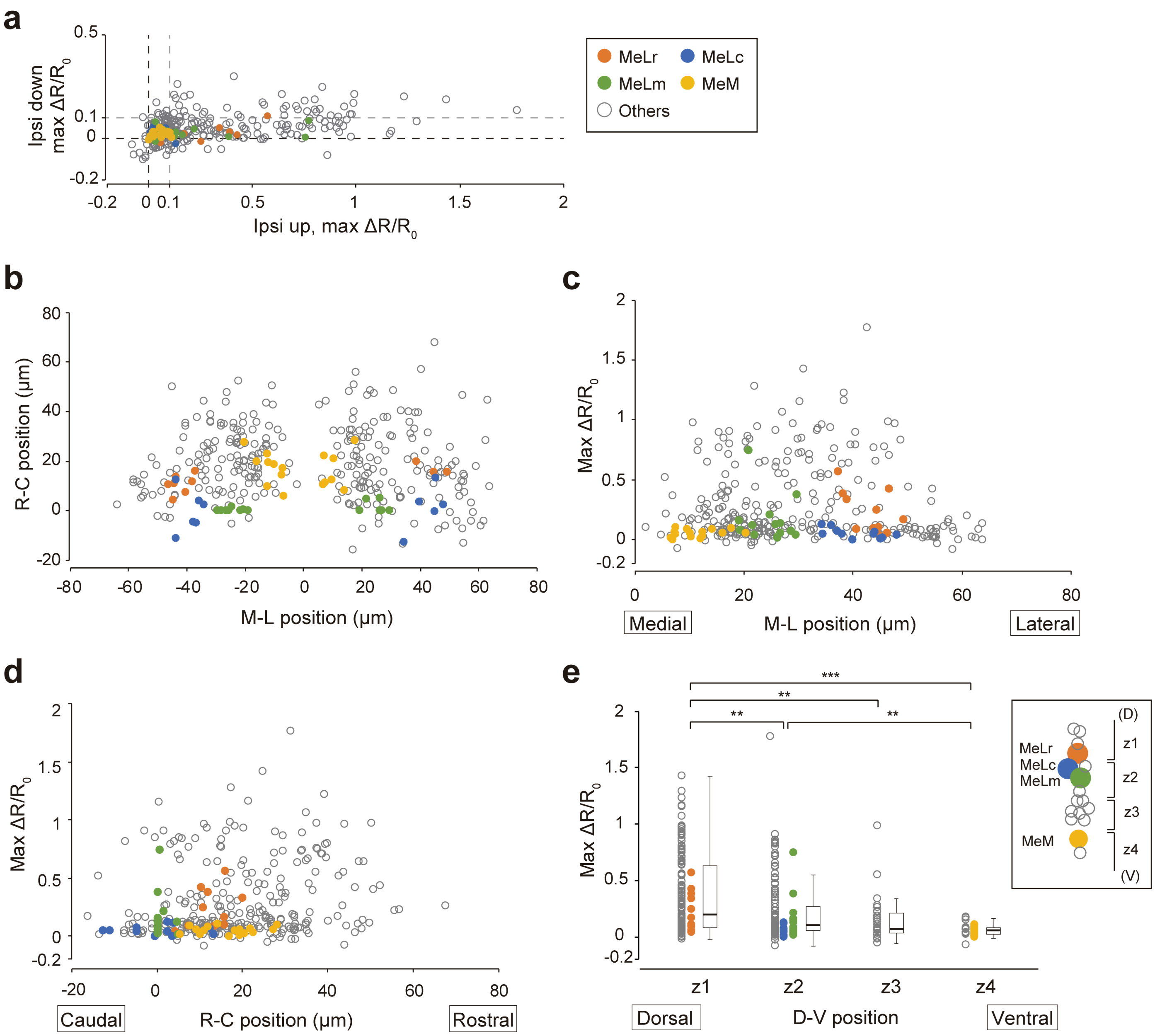

Supplementary Figure 3. Characterization of Ca^{2+} responses of nMLF neurons upon roll-tilt stimuli

Overall, 330 neurons from ten fish were analyzed. Neuronal identities (MeLr, MeLc, MeLm, MeM, and others) are shown in the right-hand side box in **a**.

a Comparison of maximum $\Delta R/R_0$ during ipsi-up and ipsi-down tilts for each neuron. The black and gray dashed lines indicate 0 and 0.1 of maximum $\Delta R/R_0$, respectively. Neurons with a maximum $\Delta R/R_0$ that was above 0.1 were judged to be active.

b Soma positions of medio–lateral and rostro–caudal axes are aligned based on the midline and the position of MeLm.

c, **d** Graphs showing the maximum $\Delta R/R_0$ during ipsi-up tilts (Y-axis) vs. the medio–lateral position (X-axis in **c**) and the rostro–caudal position (X-axis in **d**).

e Graph showing maximum $\Delta R/R_0$ during ipsi-up tilts vs. the dorso–ventral position. The dorso–ventral positions of the neurons are categorized into four groups based on their relative position to MeLr and MeM (see Methods for details of the alignment). Boxes represent the interquartile range (IQR) between the first and third quartiles and the line inside represents the median. Whiskers denote the lowest and highest values within 1.5 × IQR from the first and third quartiles, respectively. z1: n=143 cells, z2: n=130 cells, z3: n=27 cells, z4: n=30 cells z1 vs. z2: $p = 0.0057$; z1 vs. z3: $p = 0.0072$; z1 vs. z4: $p = 2.7 \times 10^{-6}$; z2 vs. z3: $p = 0.44$; z2 vs. z4: $p = 0.0028$; z3 vs. z4: $p = 0.65$ (two-sided Steel Dwass test)

Source data are provided as a Source Data file.

Supplementary Figure 4

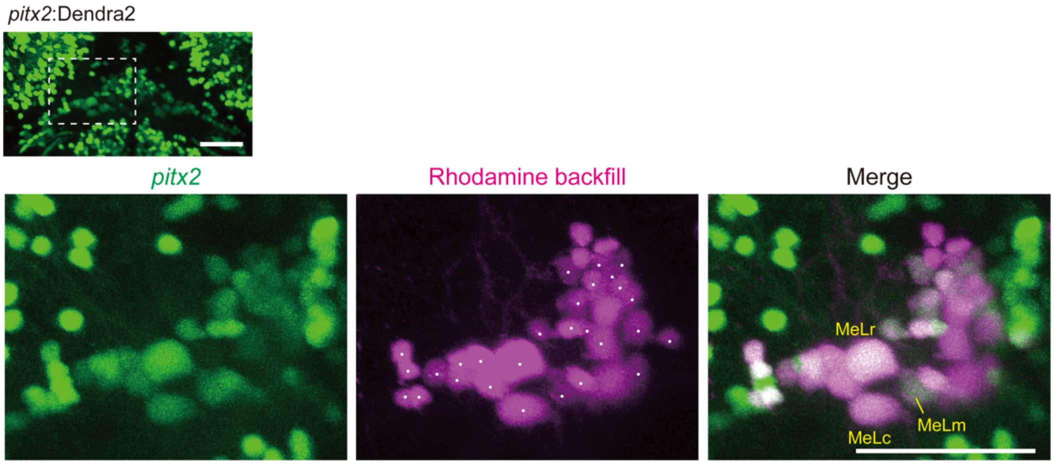

Supplementary Figure 4. Characterization of nMLF neurons labeled in Tg(*pitx2*:Dendra2) fish

Top: Confocal stacked image of Tg(*pitx2*:Dendra2). Bottom: nMLF neurons were labeled in a retrograde manner by injecting rhodamine-dextran into the rostral spinal cord. The three panels are expanded images that correspond to the white dashed box in the top panel. Dots in the middle panel indicate the rhodamine-labeled nMLF neurons that are positive for Dendra2 fluorescence. Scale bars: 50 µm

Supplementary Figure 5

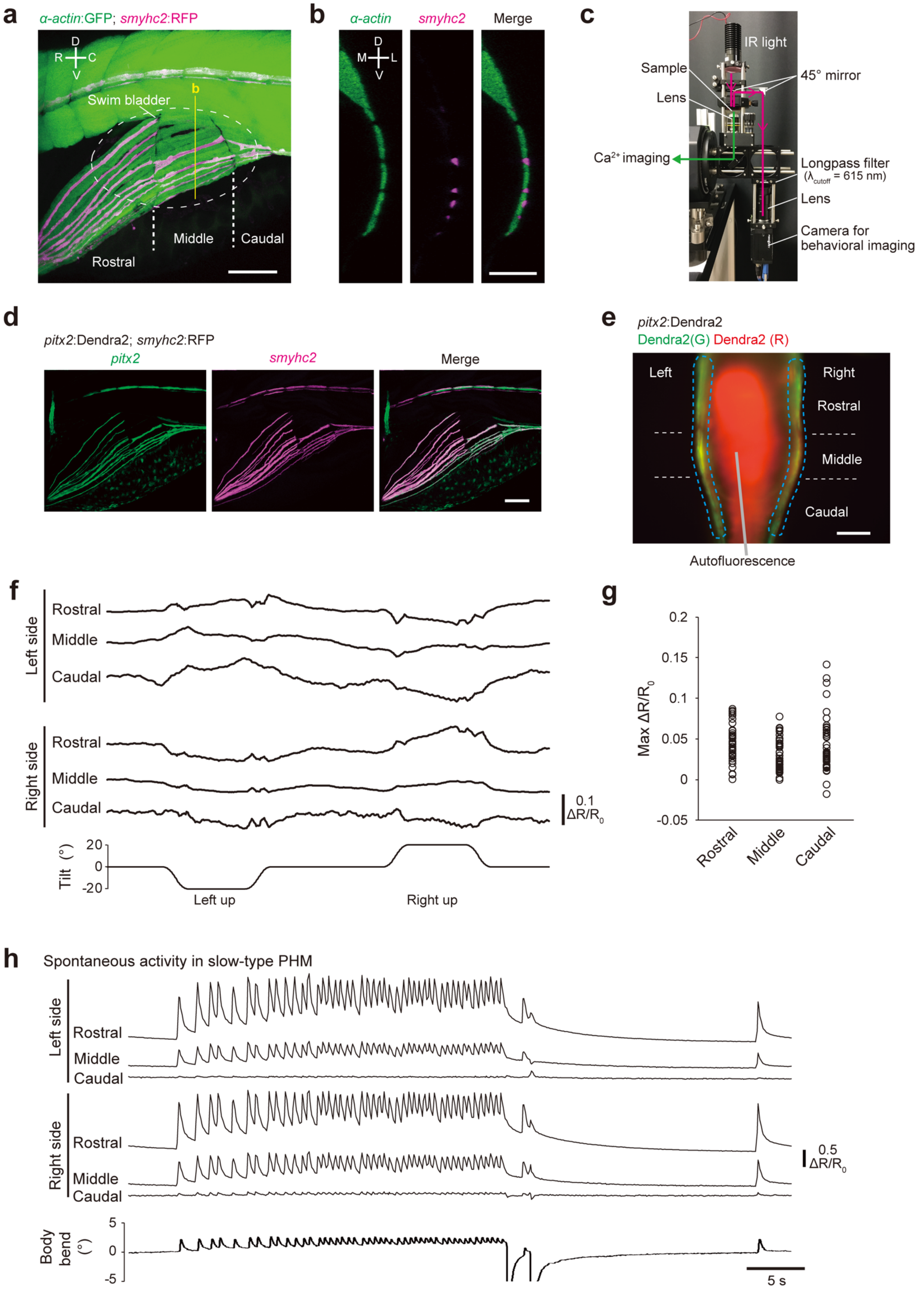

Supplementary Figure 5. Anatomical characterization of PHMs and Ca^{2+} imaging of slow-type PHMs

a Lateral view of the confocal stack image of Tg(*smyhc2*:loxP-RFP-loxP-DTA; *α-actin*:GFP) fish. PHMs consists of three segments: rostral, middle and caudal segments. White dashed circle denotes the swim bladder. R, rostral; C, caudal; D, dorsal; V, ventral.

b Cross-section view at the position of the yellow line in **a**. M, medial; L, lateral.

c Lateral view photograph of the experimental setup for both Ca^{2+} imaging and behavioral imaging. Light paths for Ca^{2+} imaging and behavioral imaging are shown in green and magenta lines, respectively.

d Lateral view of the confocal stack image of Tg(*pitx2*:Dendra2; *smyhc2*:loxP-RFP-loxP-DTA) fish. *pitx2* is expressed at slow-type PHMs.

e Image of Tg(*pitx2*:Dendra2) fish after a partial photoconversion of Dendra2. Ventral view of the area around PHMs. Green and red channels are merged. Slow-type PHMs are located at the lateral sides of fish (dashed blue lines) and are separated into three segments.

f Time course of $\Delta R/R_0$ in each segment of slow-type PHMs in response to a roll tilt.

g Maximum $\Delta R/R_0$ in each segment of slow-type PHMs during tilt. 42 dots (left and right side) in each segment, 21 trials from five fish.

h Bursts of rhythmic spontaneous activities in slow-type PHMs.

Scale bars, **a**, **d**, **e** 100 µm; **b** 50 µm

Source data are provided as a Source Data file.

Supplementary Figure 6

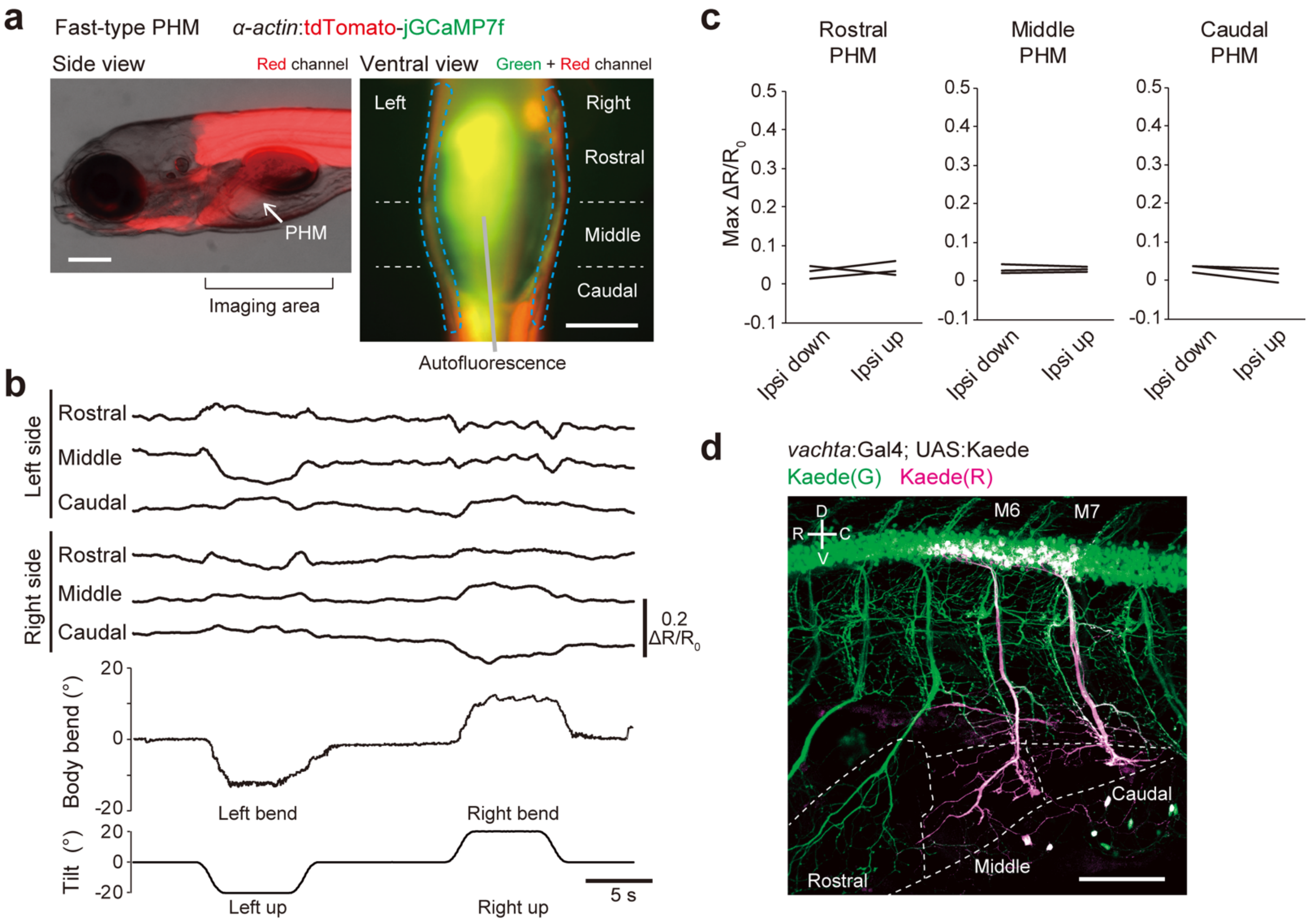

Supplementary Figure 6. Ca^{2+} imaging of fast-type PHMs and anatomical characterization of PHM motoneurons

a Fluorescent images of Tg(*α-actin*:tdTomato-jGCaMP7f) fish. Left, lateral view. Red fluorescence and transmitted light images are merged. Right, ventral view of the area around PHMs ("imaging area" in the left panel). Green and red channels are merged. Fast-type PHMs are located at the lateral sides of fish (dashed blue lines) and are separated into three segments.

b Time course of $\Delta R/R_0$ in each segment of fast-type PHMs and the body bend angle in response to a roll tilt.

c Pairwise comparison of the maximum $\Delta R/R_0$ in each segment of fast-type PHMs between ipsi-down and ipsi-up tilts (three fish). Average values of three trials are shown for each fish.

d Optical backfill (from green to red) of motoneurons innervating the middle and caudal segments of PHMs in Tg(*vachta*:Gal4; UAS:Kaede) at 5 dpf. M6 and M7 indicate the 6th and 7th muscle segments, respectively.

Scale bars, **a** 200 μm; **d** 100 μm

Source data are provided as a Source Data file.

Supplementary References

1 Kawano, K. *et al.* Long descending commissural V0v neurons ensure coordinated swimming movements along the body axis in larval zebrafish. *Sci Rep* **12**, 4348, doi:10.1038/s41598-022-08283-0 (2022).

2 Kimura, Y., Hisano, Y., Kawahara, A. & Higashijima, S.-i. Efficient generation of knock-in transgenic zebrafish carrying reporter/driver genes by CRISPR/Cas9-mediated genome engineering. *Sci. Rep.* **4**, doi:10.1038/srep06545 (2014).

3 Taniguchi, A., Kimura, Y., Mori, I., Nonaka, S. & Higashijima, S. I. Axially-confined in vivo single-cell labeling by primed conversion using blue and red lasers with conventional confocal microscopes. *Dev Growth Differ* **59**, 741-748, doi:10.1111/dgd.12412 (2017).

4 Satou, C., Kimura, Y. & Higashijima, S. Generation of multiple classes of V0 neurons in zebrafish spinal cord: progenitor heterogeneity and temporal control of neuronal diversity. *J Neurosci* **32**, 1771-1783, doi:32/5/1771 doi:10.1523/JNEUROSCI.5500-11.2012 (2012).

5 Satou, C. *et al.* Functional diversity of glycinergic commissural inhibitory neurons in larval zebrafish. *Cell Rep* **30**, 3036-3050 e3034, doi:10.1016/j.celrep.2020.02.015 (2020).

6 Satou, C. *et al.* Transgenic tools to characterize neuronal properties of discrete populations of zebrafish neurons. *Development* **140**, 3927-3931, doi:10.1242/dev.099531 (2013).

7 Higashijima, S., Okamoto, H., Ueno, N., Hotta, Y. & Eguchi, G. High-frequency generation of transgenic zebrafish which reliably express GFP in whole muscles or the whole body by using promoters of zebrafish origin. *Dev Biol* **192**, 289-299 (1997).

8 Hatta, K., Tsujii, H. & Omura, T. Cell tracking using a photoconvertible fluorescent protein. *Nat Protoc* **1**, 960-967, doi:10.1038/nprot.2006.96 (2006).
