# Supplementary material for: Biomechanics and neural circuits for vestibular-induced fine postural control in larval zebrafish
Source: Nat Commun. 2023 Mar 10;14:1217. doi: 10.1038/s41467-023-36682-y (PMC10006170; doi:10.1038/s41467-023-36682-y)
Supplement: Supplementary file 3 — Description of Additional Supplementary Files [file 41467_2023_36682_MOESM3_ESM.pdf]

File name: Supplementary Movie 1

Description: Fish behavior in response to roll tilt

Movies of the frontal and dorsal views of a fish upon a left down roll tilt. The same trial shown in Figure 1b. Frontal images are horizontally flipped (mirror-imaged) such that the left–right relationship matches with that of the dorsal image. Time course of the chamber tilt angle is shown in the left middle. After the video at the original speed, slow-motion replay from 0 s to 2 s is shown at 0.2× speed. The white arrowhead indicates the position where the VBR will occur. The magenta arrowhead indicates that the fish is performing the VBR.

File name: Supplementary Movie 2

Description: Behavior of pectoral fin-removed fish in response to a roll tilt

Same as Supplementary Movie 1, but for a pectoral fin-removed fish. The same trial shown in Supplementary Figure 1b.

File name: Supplementary Movie 3

Description: Behavior of fish immersed in methylcellulose solution in response to a roll tilt

Same as Supplementary Movie 1, but for a fish that is immersed in 0.8% methylcellulose solution. The same trial shown in Figure 1d.

File name: Supplementary Movie 4

Description: Behavior of a head-embedded fish during the roll tilts

Movie taken from the dorsal side. The video is played at 2× speed. The same trial as shown in Figure 1f.

File name: Supplementary Movie 5

Description: Behavior of swim bladder-deflated fish in response to roll tilt

Same as Supplementary Movie 1, but for a swim bladder-deflated fish. The same trial shown in Figure 2c.
