# Supplementary material for: Biomechanics and neural circuits for vestibular-induced fine postural control in larval zebrafish
Source: Nat Commun. 2023 Mar 10;14:1217. doi: 10.1038/s41467-023-36682-y (PMC10006170; doi:10.1038/s41467-023-36682-y)
Supplement: Supplementary file 9 — Reporting Summary [file 41467_2023_36682_MOESM9_ESM.pdf]

nature portfolio

Corresponding author(s): Shin-ichi Higashijima, Masashi Tanimoto

Last updated by author(s): Jan 25, 2023

Reporting Summary

Nature Portfolio wishes to improve the reproducibility of the work that we publish. This form provides structure for consistency and transparency in reporting. For further information on Nature Portfolio policies, see our Editorial Policies and the Editorial Policy Checklist.

Statistics

For all statistical analyses, confirm that the following items are present in the figure legend, table legend, main text, or Methods section.

n/a	Confirmed	
☐	☒	The exact sample size (*n*) for each experimental group/condition, given as a discrete number and unit of measurement
☐	☒	A statement on whether measurements were taken from distinct samples or whether the same sample was measured repeatedly
☐	☒	The statistical test(s) used AND whether they are one- or two-sided<br>*Only common tests should be described solely by name; describe more complex techniques in the Methods section.*
☒	☐	A description of all covariates tested
☐	☒	A description of any assumptions or corrections, such as tests of normality and adjustment for multiple comparisons
☐	☒	A full description of the statistical parameters including central tendency (e.g. means) or other basic estimates (e.g. regression coefficient) AND variation (e.g. standard deviation) or associated estimates of uncertainty (e.g. confidence intervals)
☐	☒	For null hypothesis testing, the test statistic (e.g. *F*, *t*, *r*) with confidence intervals, effect sizes, degrees of freedom and *P* value noted<br>*Give P values as exact values whenever suitable.*
☒	☐	For Bayesian analysis, information on the choice of priors and Markov chain Monte Carlo settings
☒	☐	For hierarchical and complex designs, identification of the appropriate level for tests and full reporting of outcomes
☒	☐	Estimates of effect sizes (e.g. Cohen's *d*, Pearson's *r*), indicating how they were calculated

Our web collection on statistics for biologists contains articles on many of the points above.

Software and code

Policy information about availability of computer code

Data collection	Roll tilt stimuli: Kinesis (Thorlabs, ver. 1.14.23)<br>Behavioral experiments: FlyCapture2 (Teledyne FLIR, ver. 2.13.3.61), PylonViewer (Basler, ver. 6.2.0.21487)<br>Calcium imaging: HC Image Live (Hamamatsu, ver. 4.4.0.11)<br>Confocal image acquisition: LAS X (Leica, ver. 3.5.7.23225)
Data analysis	ImageJ (NIH, ver. 1.53t)/Fiji (ver. 2.9.0), Excell (Microsoft, ver.16.65), R (R core team, ver.4.1.2)<br>For xy translation for images of calcium imaging, Template Matching and Slice Alignment Plugin (developed by Tseng Qingzong, ver. #2015/2/07) was used.<br>For image analyses, custom macro code (ImageJ/Fiji) was written. The code that support the findings of this study are available from the in Zenodo repository. The DOI is described in Code Availability section.

For manuscripts utilizing custom algorithms or software that are central to the research but not yet described in published literature, software must be made available to editors and reviewers. We strongly encourage code deposition in a community repository (e.g. GitHub). See the Nature Portfolio guidelines for submitting code & software for further information.

Data

Policy information about availability of data

All manuscripts must include a data availability statement. This statement should provide the following information, where applicable:

- Accession codes, unique identifiers, or web links for publicly available datasets
- A description of any restrictions on data availability
- For clinical datasets or third party data, please ensure that the statement adheres to our policy

The data that support the findings of this study are available as Source Data file.

Human research participants

Policy information about studies involving human research participants and Sex and Gender in Research.

Reporting on sex and gender	n/a
Population characteristics	n/a
Recruitment	n/a
Ethics oversight	n/a

Note that full information on the approval of the study protocol must also be provided in the manuscript.

Field-specific reporting

Please select the one below that is the best fit for your research. If you are not sure, read the appropriate sections before making your selection.

☒ Life sciences ☐ Behavioural & social sciences ☐ Ecological, evolutionary & environmental sciences

For a reference copy of the document with all sections, see nature.com/documents/nr-reporting-summary-flat.pdf

Life sciences study design

All studies must disclose on these points even when the disclosure is negative.

Sample size	No statistical methods were used to predetermine sample sizes. However, sample sizes are consistent with those reported in previous publications (Tanimoto et al., 2022, doi:10.1038/s41467-022-35190-9; Kawano et al., 2022, doi:10.1038/s41598-022-08283-0).
Data exclusions	In the behavioral experiments without head restraint (Fig. 1c, 2d; Supplementary Fig. 1c-h), we excluded trials in which fish performed swimming. Number of trials was between 3 and 6 per fish; if more than 6 trials without swimming were successfully collected in a fish, 6 trials were randomly selected.<br>In the head-restrained experiments, trials in which fish perform swimming during the tilts were excluded.<br>In the imaging of slow-type PHMs (Fig.8d), trials with bursts rhythmic spontaneous activity were excluded.
Replication	For all experiments, we conducted the same experiments at least 3 individuals. All replication attempts were successful.<br>We also conducted several trials per animal except for methylcellulose experiment (Figure 1d,e), nMLF imaging (Figure 6). Methylcellulose experiment was performed only once or twice because the fish fatigued in repeated trials. nMLF imaging was performed only once due to the desensitization of the calcium indicator.<br>In the behavioral experiments without head restraint, although there was a large variation in head roll and body bend angles, an overall trends were observed. In the head-restrained behavioral experiments and Ca imaging, the results were similar across the trials and animals.
Randomization	Larval zebrafish were randomly assigned to the experimental groups.
Blinding	No blinding was possible due to obvious phenotype. However, all data were analyzed with software, not scored by hand.

Reporting for specific materials, systems and methods

We require information from authors about some types of materials, experimental systems and methods used in many studies. Here, indicate whether each material, system or method listed is relevant to your study. If you are not sure if a list item applies to your research, read the appropriate section before selecting a response.

Materials & experimental systems

n/a	Involved in the study
☐	☒ Antibodies
☒	☐ Eukaryotic cell lines
☒	☐ Palaeontology and archaeology
☐	☒ Animals and other organisms
☒	☐ Clinical data
☒	☐ Dual use research of concern

Methods

n/a	Involved in the study
☒	☐ ChIP-seq
☒	☐ Flow cytometry
☒	☐ MRI-based neuroimaging

Antibodies

Antibodies used	S58 monoclonal antibody (DSHB, RRID:AB_528377, cat#: s58),<br>Alexa fluor 488-conjugated goat anti-mouse IgG secondary antibody (Thermo Fisher Scientific, RRID: AB_2534088, cat#: A11029)
Validation	S58 antibody was known to label exclusively slow myofibrils in zebrafish (ZFIN website; https://zfin.org/ZDB-ATB-081008-5).

Animals and other research organisms

Policy information about studies involving animals; ARRIVE guidelines recommended for reporting animal research, and Sex and Gender in Research

Laboratory animals	All experiments were performed on zebrafish larvae (5–6 days post fertilization) obtained from a laboratory stock of wild-type and transgenic adults.
Wild animals	This study did not involve wild animals.
Reporting on sex	Because sex is not determined at the larval stage, fish were selected randomly.
Field-collected samples	This study did not involve field-collected samples.
Ethics oversight	All procedures were performed in accordance with the guidelines approved by the animal care and use committees at the National Institute of Natural Sciences.

Note that full information on the approval of the study protocol must also be provided in the manuscript.
